# Associations among the workplace violence, burnout, depressive symptoms, suicidality, and turnover intention in training physicians: a network analysis of nationwide survey

**DOI:** 10.1038/s41598-023-44119-1

**Published:** 2023-10-05

**Authors:** Je-Yeon Yun, Sun Jung Myung, Kyung Sik Kim

**Affiliations:** 1 Department of Psychiatry, Seoul National University Hospital, Seoul, Republic of Korea; 2 Yeongeon Student Support Center, Seoul National University College of Medicine, Seoul, Republic of Korea; 3 Office of Medical Education, Seoul National University College of Medicine, 103 Daehak-ro, Jongno-gu, Seoul, 03080 Republic of Korea; 4 Department of Internal Medicine, Seoul National University College of Medicine, Seoul, Republic of Korea; 5 Department of Surgery, Yonsei University College of Medicine, Seoul, Republic of Korea

**Keywords:** Human behaviour, Network topology, Probabilistic data networks, Network models, Anxiety, Depression, Stress and resilience, Occupational health, Quality of life

## Abstract

Depression and anxiety are the most common mental disorders among physicians, who have a greater risk of suicide than those in other professional occupations. Relationships among a demanding workload, workplace violence, burnout, and intention to turnover have also been reported. The current study examined the principal components and propagating patterns of mental health and working environment interactions in training physicians. A total of 1981 training physicians completed online self-report questionnaires during September–October (midpoint of the training year) 2020. Regularized partial correlations in a mixed graphical model (MGM) and joint probability distributions (directed acyclic graph; DAG) were estimated for four subtypes of workplace violence (verbal abuse/physical violence perpetrated by clients/hospital staff), three burnout subdomains (Maslach Burnout Inventory), thoughts about quitting, and nine depressive symptoms, including suicidality, comprising the DSM-5 diagnostic criteria for major depressive disorder (assessed using the Patient Health Questionnaire-9). Thoughts of death/self-harm showed directional dependencies on the joint probability distributions of psychomotor agitation/retardation, concentration difficulty, self-reproach, and sadness in the DAG. In the MGM, a partial correlation with psychomotor agitation/retardation (r = 0.196) accounted for 56.5% of the variance in thoughts of death/self-harm. Partial correlations with concentration difficulties (r = 0.294), self-reproach (r = 0.257), changes in appetite (r = 0.184), and worker-on-worker physical violence (r = 0.240) in the MGM accounted for 54.4% of the variance in psychomotor agitation/retardation. Thoughts about quitting were partially correlated with and dependent upon the joint probability distributions of emotional exhaustion (r = 0.222), fatigue (r = 0.142), anhedonia (r = 0.178), and sadness (r = 0.237). In contrast, worker-on-worker (r = 0.417) and client-on-physician (r = 0.167) verbal abuse had regularized partial correlations with directional dependencies on thoughts about quitting. Organization-level interventions aiming to reduce the worker-on-worker violence and individual-level approaches of clinical screening program and psychiatric counseling clinic are required. Follow-up studies to verify the effectiveness of these interventions for training physicians are needed.

## Introduction

Depression and anxiety are the most common mental disorders among physicians, who have a greater risk of suicide than those in other professional occupations^1^. Burnout is associated with low job satisfaction, more patient safety incidents, lower rates of full-time work and fellowships, and a higher practice dropout rate^2–4^. In studies published up to 2018, the overall burnout rate for physicians was 67.0%, that for emotional exhaustion was 72.0%, that for depersonalization was 68.1%, and that for low personal accomplishment was 63.2%^5^. Training physicians are at greater risk of depression and burnout. The overall pooled prevalence of depression among medical residents in a meta-analysis of studies published between 1963 and 2015 was 28.8%, while the median absolute increase in depressive symptoms with the onset of residency training was 15.8%^6^. Burnout within the last year was reported by 38.5% of surgical residents^7^. Furthermore, 16.4% of medical interns^8^ and 4.5% of surgical residents^7^ reported thoughts of death or self-harm at some point during their medical training. Depressive symptoms and thoughts about death or self-harm, in addition to longer self-reported work hours and medical errors, are associated with an increased risk of suicide or self-harm during a medical internship^8^.

Work environment-related factors, such as communication problems, patient complaints, lack of support personnel, peer competition, and anxiety about the future, are putative risk factors for burnout in training physicians^4^. Feeling valued by an immediate supervisor is associated with a lower risk of burnout in training physicians^9^. Conversely, a lack of wellness resources is associated with a higher likelihood of depression and burnout in medical residents^10^. Further, stressful life experiences during the internship, in addition to the suffer of depressive symptoms and anxiety, are associated with a higher risk of developing symptoms of post-traumatic stress disorder during the final month of a medical internship^11^. First, perceived stress due to a lack of control over work (low autonomy) is related to lower self-efficacy, depressive symptoms, and burnout in medical residents during their first and second training years^4,12^. Second, possible associations have been found among the demanding workload, workplace violence, physician burnout, and turnover intention^11,13–16^. Longer work hours is an independent risk factor for anxiety, depressive symptoms, perceived stress, and burnout in medical residents^4,17–19^. Third, traumatic stress in the workplace, such as sudden patient death, serious medical errors, workplace violence, and exposure to hazards, has been reported by 56.4% of medical interns^11^. Notably, workplace violence is a major occupational issue for medical professionals, and has a significant effect on the physical and psychological well-being of physicians^13^. The overall prevalence of patient/client-on-physician workplace violence ranges from 9.5 to 74.6%, including verbal abuse (42.1–94.3%), threats of assault (14.0–57.4%), bullying (2.5–5.7%), and physical violence (0.5–15.9%)^20^. Verbal abuse, physical violence, and sexual harassment by fellow workers are reported by 13–30.2%, 2.2–7.1%, and 10.3–19.9% of medical residents, respectively^7,19,21–23^. Exposure to workplace violence has been associated with reduced work performance, anger, burnout, depression, and thoughts about quitting^7,16,19,20,22^.

Phenotypic networks^24–27^ can be used to delineate the relationships among depressive symptoms, burnout, thoughts about quitting, and workplace violence in physicians during a medical internship or residency. MGMs^28^ calculate the parameterized joint probability density among variables^29^. MGMs have been used to examine the regularized partial correlations between the symptoms of major depression and stressful life events, childhood abuse, and neuroticism^30^; between thoughts about death/self-harm and feelings of guilt, sadness, and other non-depressive psychological symptoms^31,32^, and between sleep disturbances and fatigue and serum markers of inflammation^25^. Undirected item-to-item interactions can be detected based on the cooccurrence or covariance of nodes (or features) in the MGM^25^. Further, deriving local graph theory metrics, such as betweenness centrality, for the nodes comprising the MGM can reveal the principal components or hubs^33^. Network hubs reflect trends in symptom status in a given network and may reveal candidate therapeutic targets for interventions^33^. DAGs^24^ display the joint probability distributions of conditional dependencies, such as the COVID-19 pandemic and verbal abuse, depressive and anxiety symptoms, cognitive style and behavioral patterns, and self-esteem and social interactions^27,34^. As the presence of a downstream component “B” next to the directional arrow implies the upstream occurrence (before the arrowhead in the DAG) of component ”A” in the DAG, the DAG phenotypic network could be useful for estimating the propagating patterns of psychological symptoms and symptom-behavior associations^35^. The complementary use of MGMs and DAGs may be useful for revealing the most important items within a network by deriving centrality values and patterns of psychological symptoms or environmental factors based on the pattern of arrows in the network.

Detailed information about the associations^27^ among workplace violence subtypes, burnout subtypes, turnover intention, and depressive symptoms, including suicidality, must be elucidated so that interventions can effectively reduce exposure to, and the harmful effects on mental health of, workplace violence among training physicians^11^. Of note, depressive symptoms not only coexist in mood episodes but also demonstrate inter-item variations in the duration and intensity^36,37^. Sadness and anhedonia are associated with a 3.4–5.1-fold increased risk of burnout in emergency medicine medical residents^38^. In addition, the negative coping strategies of self-blame, venting, and substance use are associated with a higher likelihood of burnout in medical residents and fellows working on the frontline of the COVID-19 pandemic^9^. However, depression is often described as a single construct rather than examining the specific symptoms that comprise the DSM-5 diagnostic criteria for major depressive disorder such as anhedonia, psychomotor agitation/retardation, changes in appetite, self-reproach, and suicidality. On the other hand, a MGM-based network approach of medical professionals demonstrated that burnout symptoms could be segregated from depressive and anxiety symptoms^39^. However, few studies have revealed the principal components and multiple key connections among mental health- and work environment-related factors in training physicians^26,27,33,34^.

Therefore, in the current study, the principal components and propagating patterns of mental health-working environment interactions in training physicians were identified using a phenotypic network approach. Regularized partial correlations (MGM) and joint probability distributions (DAG) were estimated among four subtypes of workplace violence (verbal abuse and physical violence perpetrated by clients or other physicians), thoughts about quitting, three subdomains of burnout (emotional exhaustion, depersonalization, and personal achievement), and the nine depressive symptoms, including thoughts of death/self-harm, comprising the DSM-5 diagnostic criteria for major depressive disorder^40^. Specifically, based on previous studies that demonstrated differential associations between item-level depressive symptoms and other psychopathological and environmental factors^27,41–43^, the nine depressive symptoms comprising the DSM-5 diagnostic criteria for major depressive disorder were retained as separate nodes in the network analyses in the current study. Also, in consideration of previous studies for physicians that reported heterogeneity in associations of sex with depression^6^, burnout^5^, workplace violence^11,44^, turnover intention^45^, and suicidality^46^, we performed between-group comparisons of MGM and DAG between male and female physicians. As study hypotheses, we expected to find connections of workplace violence, burnout, thoughts about quitting, depression, and thoughts about death or self-harm with depressive symptoms such as psychomotor retardation/agitation, feelings of worthlessness, and emotional exhaustion in the MGM^47,48^. Second, we hypothesized that, in the DAG, psychomotor changes and feelings of worthlessness would be conditionally dependent on thoughts about death/self-harm^49,50^. Third, a joint probability distribution was expected among emotional exhaustion, reports of workplace violence, and thoughts about quitting.

## Methods

### Participants

The current study was conducted between 23 September 2020 and 14 October 2020 and used self-report questionnaires. After obtaining a license to practice as a physician from the Ministry of Health and Welfare (https://www.mohw.go.kr/eng/), almost all new medical doctors undertake further training at training hospitals to become medical specialists. A 1-year internship and 4-year residency in a specialty (3-year residency for internal medicine, general surgery, pediatrics, and family medicine), which typically starts in March and ends in February of the next year, are required to apply for the test to qualify as a medical specialist. September and October are in the middle of the medical training year, which starts in March, and midyear is well suited for assessing training physician mental health-working environment interactions.

This study included medical interns or residents affiliated with training hospitals in the Republic of Korea, who were members of the Korean Intern Resident Association (KIRA) as of September 2020 (N = 13,571) and were willing to voluntarily participate in our web-based survey during September–October 2020. Promotional documents were posted on the KIRA website (http://youngmd.org/). After reading the promotional documents, the medical intern or resident clicked the link for the online survey. Informed consent was indicated by clicking the “Start” button on the front page of the Google survey. The survey was anonymous, and confidentiality was ensured. Study participation was on a first-come, first-served basis during the 3-week data acquisition period, and 1981 (14.6% of the total population) of medical interns and residents took part. Participants were given coffee vouchers worth 4 USD as remuneration. This study was approved by the Clinical Research Ethics Committee of Seoul National University College of Medicine and Hospital (IRB No. 2202-019-1296). All procedures followed the relevant guidelines and regulations.

### Measures

The online self-report questionnaire used in this study was about four A4 pages long and took 5–10 min to complete. Demographic data (sex, training stage, and weekly work hours) and information about workplace violence (number of incidents and perpetrators of verbal abuse, physical violence, or sexual harassment), burnout [emotional exhaustion, depersonalization, and personal achievement, measured using the Maslach Burnout Inventory (MBI)^51,52^], turnover intention, and depressive symptoms, including suicidality [sadness, anhedonia, insomnia, appetite change, fatigue, self-reproach, psychomotor changes, concentration difficulty, thoughts of self-harm or suicide, measured using the Patient Health Questionnaire-9 (PHQ-9)^53–55^] were acquired. The internal consistency of the MBI data was good (Cronbach's alpha = 0.856), and that of the PHQ-9 data was excellent (Cronbach's alpha = 0.916). The Cronbach’s alpha values were calculated using the c*ronbach.alpha* function in the R package *ltm* (https://rdrr.io/cran/ltm/)*.* Appropriate monitoring of participant responses was ensured during this web-based survey.

Reports of verbal abuse, physical violence, and sexual harassment in the workplace within the past 4 months were recorded in terms of the number of incidents (0, 1, 2, 3, 4, or ≥ 5 events) and the perpetrators [patient(s)/caregiver(s), physician(s), and other hospital staff; multiple responses permitted]. Burnout was measured using a version of the MBI validated for use in Korea^51,52^. We purchased the MBI license before administering the instrument via the MindGarden (http://www.mindgarden.com) webpage. The 22 items of the MBI are scored using a 7-point Likert-type scale (0, not at all; 1, a few times per year; 2, once a month; 3, a few times per month; 4, once a week; 5, a few times per week; 6, every day). The cut-off scores for the burnout subdomains followed previous Korean studies, as follows: emotional exhaustion, ≥ 27; depersonalization, ≥ 10; and personal accomplishment, ≤ 33^56,57^. Higher scores reflect higher levels of emotional exhaustion (9 items) and depersonalization (5 items), and lower scores reflect lower levels of personal achievement (8 items, including reverse-coded ones)^58^. Thoughts about quitting medical training were measured using a single item (“During the past 4 months, have you ever thought about quitting?”) that required a “yes” or “no” response. Depressive symptoms were measured using the version of the PHQ-9 validated for use in Korea^53–55^. Responses to the PHQ-9 items were scored on a 4-point Likert scale (0, not at all; 1, 2–6 days; 2, 7–12 days; 3, nearly every day). Total PHQ-9 scores range between 0 and 27. The cut-off for moderate depressive mood was a total PHQ-9 score ≥ 10, as reported previously for the Korean population^55,59,60^. The response to item 9 of the PHQ-9 was used to determine the presence (answer = 1–3) or absence (answer = 0) of suicidality.

### Network analyses

Multiple associations among the nine depressive symptoms of major depressive disorder (including thoughts of death/self-harm) in the DSM-5 (PHQ-9), three burnout subdomains (MBI), thoughts about quitting, and four subtypes of workplace violence (physical violence/verbal abuse [client-on-physician/worker-on-worker]) during medical training were examined using MGM^28^ and DAG^61^ network analyses. The nodes were selected based on previous research. First, depressive symptoms^38^, including thoughts of death/self-harm^7^, are associated with an increased risk of burnout. Second, burnout is associated with increased thoughts about quitting^2–4^. Third, perceived workplace violence may be associated with reduced work performance, anger, burnout, depression, and thoughts about quitting^7,16,19,20,22^. In these networks, variables are nodes (M = 15 for the current study), and “edges” reflect undirected (MGM)^61^ and directional (DAG)^25^ joint probability distributions of conditional dependencies among nodes.

First, the MGM estimates regularized partial correlation coefficients, which represent edge weights in node-wise regression^31^, using the R-package *mgm*^28^*.* The thickness of each edge in the MGM represents the strength of the association, with thicker edges representing stronger associations^62^. Of note, aiming to remove potentially spurious associations and to ensure a more robust network structure, a penalty based on the least absolute shrinkage and selection operator (LASSO)^63^ was applied to shrink the edge weights and to reduce smaller edge weights to zero. For finding the optimal MGM structure, we also utilized a pairwise model (interaction order k = 2) and the extended Bayesian information criterion (tuning parameter γ = 0)^25^. Variables that were not connected when conditioned on other variables were considered independent^64^. To identify the principal nodes reflecting trends in mental health-working environment interactions within a given MGM network^33^, a local graph metric of betweenness centrality (proportion of all short paths in the network that contain a given node)^65^ was calculated. Two nodes ranked in the top 12% for betweenness centrality were defined as hubs^26,27,43^. R^2^ regression values were estimated for each node to quantify how well it was predicted by other nodes with which it shared an edge^25,66,67^. These “node predictability values” are represented as pie charts^66^. The MGM network structure was visualized as a circular layout using the R-package *qgraph*^62^.

Second, the DAG provides a probabilistic graphical model (Bayesian network) that represents the joint probability distribution of conditional dependencies (shown by directed and noncircular edges) among the variables (or nodes). By applying the hill-climbing greedy search algorithm using the R package *bnlearn*^68^*,* the DAG added edges (connecting different variables or nodes), and then removed them/reversed their direction until the BIC goodness-of-fit cut-off was reached^42^. Whether an edge exists between two variables was determined by an iterative procedure with 10,000 bootstraps^42,69^. The average network structure was determined by retaining the dominant edges among the 10,000 bootstrapped networks, according to a statistically determined cut-off point; edges with high sensitivity and specificity were retained^42,70^. Finally, BIC values were computed for each edge, with higher values signifying greater importance within the network structure^42^.

**Figure 1:**
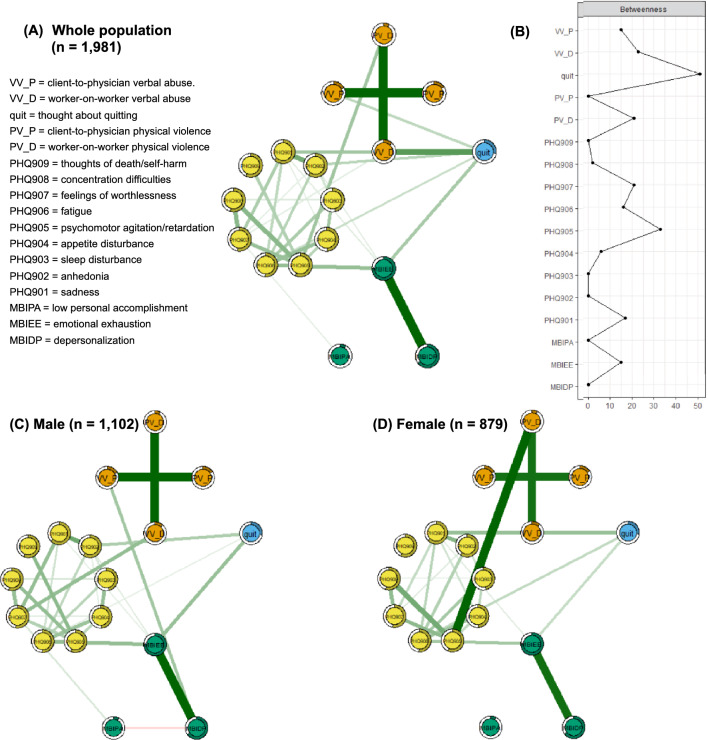
(**A**) Mixed graphical model (MGM) of the undirected partial correlations among the frequency of item-level depressive symptoms including thoughts about death/self-harm, severity of burnout subtypes (depersonalization, emotional exhaustion, and lower personal accomplishment), thought about quitting, and workplace violence (physical violence and verbal abuse; patient/client-to-physician and worker-on-worker) in medical interns and residents (n = 1981). The “node predictability value” (variance in a given node’s value explained by the nodes with which it is connected) is indicated by the shadowed parts of rings surrounding each node. (**B**) Betweenness centrality (the proportion of the shortest paths in the network containing a given node) was used as the centrality measure. The x-axis represents z-scores). (**C**) The MGM model of males (n = 1102). (**D**) The MGM model of female (n = 879). PHQ901: sadness; PHQ902: anhedonia; PHQ903: sleep disturbance; PHQ904: appetite disturbance; PHQ905: psychomotor agitation/retardation; PHQ906: fatigue; PHQ907: feelings of worthlessness; PHQ908: concentration difficulties; PHQ909: thoughts about death or self-harm; MBIEE: emotional exhaustion; MBIDP: depersonalization; MBIPA: low personal accomplishment; quit: thought about quitting the medical internship or residency; PV_D: worker-to-worker physical violence; PV_P: physical violence perpetrated by a patient/client; VV_D: worker-to-worker verbal abuse; VV_P: patient/client-to-physician verbal abuse.

**Figure 2:**
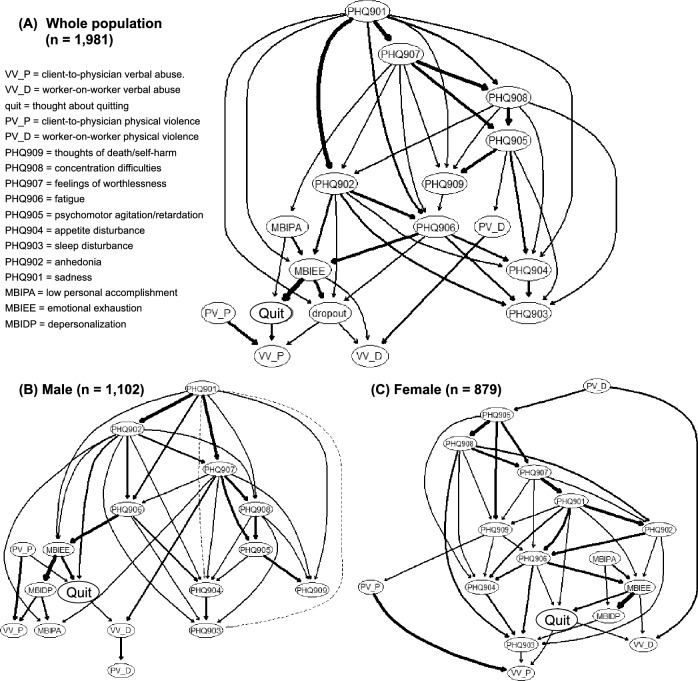
(**A**) Directed acyclic graph (DAG) showing the relationships among the frequency of item-level depressive symptoms including thoughts about death/self-harm, severity of burnout subtypes (depersonalization, emotional exhaustion, and lower personal accomplishment), thought about quitting, and workplace violence (physical violence and verbal abuse; patient/client-to-physician and worker-on-worker) in medical interns and residents (n = 1982). (**B**) The DAG model of males (n = 1102). (**C**) The DAG model of female (n = 879). PHQ901: sadness; PHQ902: anhedonia; PHQ903: sleep disturbance; PHQ904: changed appetite; PHQ905: psychomotor agitation/retardation; PHQ906: fatigue; PHQ907: self-reproach; PHQ908: concentration difficulties; PHQ909: thoughts about death or self-harm; MBIEE: emotional exhaustion; MBIDP: depersonalization; MBIPA: low personal accomplishment; quit: thought about quitting the medical internship or residency; PV_D: worker-to-worker physical violence; PV_P: physical violence perpetrated by a patient/client; VV_D: worker-to-worker verbal abuse; VV_P: patient/client-to-physician verbal abuse.

In addition, MGM subgroup estimates were determined for males (n = 1,102; Fig. 1C) and females (n = 879; Fig. 1D) separately. Invariance between the MGM of males (n = 1,102) and MGM of females (n = 879) was tested using the permutation test (number of permutations = 10,000) and the *NetworkComparisonTest* (NCT) toolbox in R software^71,72^. We tested for differences in global strength, network structure invariance, and the invariance of betweenness centrality values between the networks. Additional DAGs were generated for subgroups of males (n = 1,102; Fig. 2B) and females (n = 879; Fig. 2C) separately.

### Role of the funder

The funders of the study had no role in study design, data collection, data analysis, data interpretation, or writing of the report. The corresponding author had full access to all the data in the report and had final responsibility for the decision to submit for publication.

## Results

### Sample characteristics

Table 1 illustrates the demographic, work-related, and clinical characteristics of the study participants. A total of 1,981 medical interns and residents participated in this study (1102 males and 879 females). There were 490 (24.7%) medical interns, 354 (17.9%) first-year residents, 354 (17.9%) second-year residents, 411 (20.7%) third-year residents, and 372 (18.8%) fourth-year residents. A total of 1365 (68.9%) participants worked for ≤ 80 h per week, and 616 (31.1%) worked for > 80 h. Exposure to verbal abuse, physical violence, and sexual harassment within the past 4 months was reported by 482 (24.3%), 77 (3.9%), and 100 (5.0%) participants, respectively. Emotional exhaustion (MBI subscale score ≥ 27), depersonalization (MBI subscale score ≥ 10), and low personal achievement (MBI subscale score ≤ 33) were present in 771 (38.9%), 813 (41.0%), and 1,627 (82.1%) participants, respectively. A total of 1,049 (53.0%) participants reported that they thought about quitting medical training. Depression (total PHQ-9 score ≥ 10) and thoughts of death or self-harm (item 9 on the PHQ-9 ≥ 1) were reported by 323 (16.3%) and 172 (8.7%) participants, respectively.

**Table 1:** Demographic, work-related, and clinical characteristics.

Variables		N (% of study population)			
Sex (M/F)		1102 (55.6%)/879 (44.4%)			
Training stage	Medical intern	490 (24.7%)			
	1st year residents	354 (17.9%)			
	2nd year residents	354 (17.9%)			
	3rd year residents	411 (20.7%)			
	4th year residents	372 (18.8%)			
Perceived workplace violence within the recent 4 months: YES	Verbal abuse	482 (24.3%)			
	Physical violence	77 (3.9%)			
	Sexual harassment	100 (5.0%)			
Thought about quitting the medical training: YES		1,049 (53.0%)			
Thought of death/self-harm (9th item of PHQ-9 ≥ 1)		172 (8.7%)			

Variables		M ± SD	Range	Skewness	Kurtosis
Weekly workhours		77.5 ± 17.9	6–168	− 0.233	4.596
Maslach Burnout Inventory	Emotional exhaustion	23.8 ± 14.4	0–60	0.448	2.225
	Depersonalization	8.9 ± 7.4	0–30	0.638	2.457
	Low personal achievement	21.7 ± 11.6	0–42	− 0.298	2.104
Total score of the Patient Health Questionnaire-9		4.8 ± 5.7	0–27	1.72	6.012

### Network analyses: network structures, hub nodes, and key edges of the MGM and DAG

The MGM or regularized undirected partial correlations among the nine depressive symptoms of major depressive disorder (including thoughts of death/self-harm) in the DSM-5, three burnout subdomains, thoughts about quitting, and four subtypes of workplace violence during medical training are presented in Fig. 1A. The pie charts surrounding each node^66^ in Fig. 1A depict the node predictability (R^2^)^67^, which is the predictability of each node by nodes connected via edges in the MGM. In contrast, the DAG shown in Fig. 2A estimates the propagating patterns among the nodes based on cross-sectional data^24^. The presence of downstream component B next to the directional arrow implies the upstream (before the arrowhead in the DAG) occurrence of component A.

Sadness had the highest node predictability value, of 62.3%, in the MGM and was the most upstream component in the DAG. Self-reproach was dependent on sadness and was associated with all other depressive symptoms, except sleep disturbance and appetite changes, in the DAG. The burnout subdomain of low personal accomplishment was dependent on self-reproach. Among the three burnout subdomains, node predictability was higher for emotional exhaustion (74.7%) and depersonalization (65.1%) than personal accomplishment (8.2%). Client-on-physician physical violence had no parental node in the DAG and was associated with client-on-physician verbal abuse. As indicated by their higher betweenness centrality values (Fig. 1B), thoughts about quitting (z-score = 2.65) and psychomotor agitation/retardation (z-score = 1.40) were the components/mediators most frequently included in the shortest paths connecting different nodes in the MGM. Partial correlations and directional dependence of emotional exhaustion and fatigue (r = 0.264), and of worker-on-worker physical violence and psychomotor agitation/retardation (r = 0.240), were the strongest mediators of the relationships of depressive symptoms with burnout and workplace violence, respectively. Worker-on-worker verbal abuse and emotional exhaustion were not independent, but they were conditionally independent on thoughts about quitting in the MGM and DAG.

### Network analyses: factors associated with thoughts about quitting and death/self-harm

The node predictability of thoughts about quitting was 26.7% in the MGM, based on the partial correlations with sadness (r = 0.237), anhedonia (r = 0.178), fatigue (r = 0.142), emotional exhaustion (r = 0.222), worker-on-worker verbal abuse (r = 0.417), and client-on-physician verbal abuse (r = 0.167) was 26.7% in the MGM. According to the information on directionality in the DAG, thoughts about quitting were dependent upon the joint probability distributions of emotional exhaustion, fatigue, anhedonia, and sadness. Conversely, perceived worker-on-worker verbal abuse (node predictability in the MGM = 12.2%) had regularized partial correlations with, and was dependent on, the joint probability distributions of worker-on-worker physical violence (r = 2.079), thoughts about quitting (r = 0.417), and emotional exhaustion (self-reproach in MGM; r = 0.076). Client-on-physician verbal abuse (node predictability in MGM = 17.1%) had regularized partial correlations with and directional dependence on the joint probability distributions of client-on-physician physical violence (r = 2.262), depersonalization (no association in MGM), and thoughts about quitting (r = 0.167).

A node predictability value of 56.5% was calculated for thoughts of death/self-harm in the MGM based on the partial correlation with psychomotor agitation/retardation (r = 0.196). Psychomotor agitation/retardation also had partial correlations with concentration difficulties (r = 0.294), self-reproach (r = 0.257), changes in appetite (r = 0.184), and worker-on-worker physical violence (r = 0.240; node predictability in the MGM = 7.8%). Directional dependencies of thoughts of death/self-harm on the joint probability distributions of psychomotor agitation/retardation, concentration difficulty, self-reproach, and sadness were found in the DAG. In contrast, thoughts of death/self-harm in the DAG had a directional association with fatigue (not found in the MGM).

### Network analyses: network comparisons of males and females

Network characteristics were compared between the MGM of males (n = 1102; Fig. 1C) and the MGM of females (n = 879; Fig. 1D) using the NCT toolbox in R software^71,72^. The permutation test with the NCT (number of permutations = 10,000) did not detect a significant difference in global strength (i.e., the sum of all edge weights in a network^73^; P = 0.588) between the MGM of males (global strength = 7.013) and that of females (global strength = 7.223). Additionally, the test for network structure invariance (i.e., differences or similarities between two networks in terms of the structure of node relationships^74^; P = 0.256) and possible differences in betweenness centrality values (all *P*s > 0.05; FDR-corrected) between the MGMs of males and females were not significant.

The directional conditional dependence of thoughts of death/self-harm on the joint probability distributions of sadness, self-reproach, concentration difficulties, and psychomotor agitation/retardation found in the original DAG (Fig. 2A; n = 1981) were preserved in the DAGs for the subgroups of males (Fig. 2B; n = 1102) and females (Fig. 2C; n = 879). The chains of associations from anhedonia to thoughts about quitting, and from thoughts about quitting to perceived worker-on-worker verbal abuse, seen in the original DAG of the entire population (Fig. 2A; n = 1981) were replicated in the DAGs for the subgroups of males (Fig. 2B; n = 1102) and females (Fig. 2C; n = 879).

## Discussion

### Study summary

The current study examined the principal components and propagating patterns of mental health-workplace violence interactions in training physicians using an MGM (Fig. 1A) and DAG (Fig. 2A). For estimating the more robust MGM structure without spurious false-positive edges, a LASSO^63^-based penalty approach shrunk the edge weights (= regularized partial correlations) and reduced smaller edge weights to zero. Thoughts of death/self-harm in the MGM had a partial correlation with psychomotor agitation/retardation (r = 0.196), which accounted for 56.5% of the variance in thoughts of death/self-harm (having a node predictability value of 0.565 in the MGM). In turn, partial correlation of psychomotor agitation/retardation with concentration difficulties (r = 0.294), self-reproach (r = 0.257), changes in appetite (r = 0.184), and worker-on-worker physical violence (r = 0.240) could account for 54.4% of the variance in psychomotor agitation/retardation (with a node predictability value of 0.544 in the MGM). Thoughts of death/self-harm demonstrated directional dependencies on the joint probability distributions of psychomotor agitation/retardation, concentration difficulty, self-reproach, and sadness in the DAG. Thoughts about quitting were partially correlated with and dependent upon the joint probability distributions of emotional exhaustion (r = 0.222), fatigue (r = 0.142), anhedonia (r = 0.178), and sadness (r = 0.237). In contrast, worker-on-worker and client-on-physician verbal abuse had regularized partial correlations with directional dependencies on thoughts about quitting (r = 0.417 and 0.167, respectively). All these six edges of partial correlations could account for 26.7% of the variance in thought about quitting (with a node predictability value of 0.267 in the MGM).

### Psychomotor changes underlie thoughts about death or self-harm in training physicians

Which components of depressive symptoms, workplace violence, and burnout are most strongly associated with thoughts about death/self-harm, even after regressing out the effects of other items? The answer was revealed by our MGM (Fig. 1A). The regularized partial correlation between thoughts about death/self-harm and psychomotor changes explained 56.5% of the variance in thoughts about death/self-harm. This result follows those of a previous study in which thoughts about death or self-harm and psychomotor agitation/retardation improved in response to antidepressants (sertraline and/or mirtazapine)^75^. How did the activation of item-level depressive symptoms influence thoughts about death/self-harm? Although we cannot infer causality from the cross-sectional data, the presence of a sibling node after the directional arrow suggests the presence of a parent node before the directional arrow in the DAG (Fig. 2). Therefore, we presumed that sadness, feelings of worthlessness, concentration difficulties, and psychomotor changes were present in training physicians who experienced thoughts about death or self-harm. These results follow those of other studies demonstrating close associations among thoughts about death/self-harm, psychomotor agitation/retardation^76^, and feelings of worthlessness in phenotypic networks^77^. Feelings of worthlessness, psychomotor retardation, thoughts about death or self-harm, and psychotic ideation over 6 months can result in role impairment, even in cases of subthreshold depression^78^. Similarly, depressive individuals with thoughts about death/self-harm and impaired executive control and decision-making may change their behavior, for example showing increased risk-taking, poor impulse control, and psychomotor agitation^79^. Access to and knowledge of effective lethal methods of suicide^80,81^, in addition to insufficient time to access mental healthcare^82^, may be associated with the increased risk of suicide seen in physicians relative to non-physicians. Multifaceted interventions combining individual- and organizational-level approaches are required to reduce the risk of physician suicide^1^ and the multiple, interrelated problems found in cases thereof^81^. First, pharmacotherapy to reduce psychomotor agitation^83,84^ and cognitive behavioral approaches to anti-suicidal therapy^85–87^, including modifying hopelessness and feelings of worthlessness^88,89^ are required. Second, organizational interventions to improve work-related risk factors for poor physician mental health, such as work-life imbalance, excessive or conflicting job demands, longer working hours, and interpersonal conflict are important^90–92^.

### Emotional exhaustion, anhedonia, fatigue, and worker-on-worker verbal abuse underlie thoughts about quitting

Previous studies on hospital staff, including physicians, have reported that a large number of weekly work hours^93^, monthly night shifts^93,94^, lower average monthly income^16,94^, workplace violence^16,93–96^, burnout^93,97^, and depressive symptoms^16,94^ are associated with thoughts about quitting. Notably, the current study was performed during the COVID-19 pandemic, which imposed severe stress on healthcare systems and economies worldwide, including those of the Republic of Korea. The psychological impacts of the pandemic promoted nervousness and stress in daily life, trouble relaxing, and feelings of failure, followed by trouble concentrating, loss of situational control, and fear of infecting colleagues^34^. Second, worsening mental and social health in people with lower monthly household incomes or education levels became more likely during the COVID-19 pandemic compared to the pre-pandemic era^98^. Healthcare providers in South Korea had to work in critical situations and were overwhelmed by heavy workloads, fear of infection, lifestyle changes, and psychological and physical struggles during the COVID-19 pandemic^99^. Environmental (insufficient personal protective equipment, working in a screening center, prolonged work hours) and psychosocial (fear of infection and death, social stigma, and rejection) aspects of work were associated with poor mental health in South Korean public health physicians on the frontline. In contrast, satisfactory monetary compensation and proactive coping (acceptance and willingness to volunteer on the frontline) were predictive of better mental health^100^. In the MGM of the current study (Fig. 1), the regularized partial correlations of thoughts about quitting with worker-on-worker verbal abuse, emotional exhaustion, sadness, anhedonia, and fatigue explained 26.7% of the variance in thoughts about quitting. This result follows other phenotypic network analyses demonstrating that depressive symptoms^101^, including high self-efficacy (a feeling of invulnerability)^102,103^, anxiety about violence^101,104^, burnout^105^, and perceived social^101^ and organizational^93,102^ support mediate the relationship between workplace violence and thoughts about quitting.

The current results demonstrate the importance of interventions^106–108^ targeting both individual^109^ and organizational^110^ risk factors for thoughts about quitting. The DAG (Fig. 2) implied the presence of anhedonia, fatigue, and emotional exhaustion in training physicians who reported thoughts about quitting. To prevent and remediate emotional exhaustion and intention to quit in training physicians, an organizational approach involving clinical assessment programs targeting emotional exhaustion, sadness, anhedonia, and fatigue is required. It is also necessary to operate a psychiatric counseling clinic for training physicians and establish an administrative system that enables them to schedule visits to the outpatient clinic during the day. Our DAG (Fig. 2) also indicated that worker-on-worker verbal abuse suggests the presence of thoughts about quitting in training physicians (Fig. 2). To prevent and remediate verbal abuse (worker-on-worker and client-on-physician), organizational interventions are paramount. First, organizational policies of cultural reforms particularly in the context of reducing worker-to-worker aggression and violence are required^111^. Second, organizational allocation of sufficient physician manpower to understaffed workplace is required to prevent understaffing-related excessive job demands and workplace aggression/violence^112,113^. Third, organizational-level prompt intervention of workplace violence occurrence by running of simulation-based behavioral training^114^ and operating of behavioral emergency response teams^115^ are needed. Furthermore, proactive cognitive-behavioral interventions to deal with verbal abuse, as well as preventive screening toolkits combined with one-step psychiatric consultations, are required^116^.

### Strengths and limitations

The current study has strengths such as relatively large sample size (N = 1981), use of comprehensive instrument battery, and complementary use of both MGM and DAG in network analyses. Also, this study has some limitations. First, it used a cross-sectional design, so causality cannot be inferred. Second, as study participation was on a first-come, first-served basis during the 3-week data acquisition period, representativeness of the study sample for total population cannot be assured. Third, the workplace violence data for the 4-month study period were based on participant recall. Prospective cohort studies may be more suitable, as they minimize recall bias. Fourth, possible protective factors against work-related stress, such as life satisfaction^26^, were not investigated. We limited the length of the survey to reduce participant burden, but could not prioritize measurement of protective factors over mental health and work environment-related factors. Additional phenotypic network analyses of work-related stress, mental health, and other protective and risk factors for thoughts about quitting are required using longitudinal follow-up data and larger sample sizes.

## Conclusions

This study used an MGM and DAG to examine the principal components and propagating patterns of mental health-workplace violence interactions in training physicians. Of note, partial correlation with psychomotor agitation/retardation could account for 56.5% of the variance in thoughts of death/self-harm. Moreover, partial correlations with concentration difficulties, self-reproach, changes in appetite, and worker-on-worker physical violence could account for 54.4% of the variance in psychomotor agitation/retardation. Follow-up studies to examine the longitudinal stability of current study findings and the effectiveness of organizational- and individual-level interventions in reducing the workplace violence, thought about quitting, and suicidality of training physicians are required. Most of all, organization-level interventions of cultural reforms aiming to reduce the worker-to-worker aggression/violence, allocation of sufficient physician manpower to prevent the understaffing-related workplace aggression/violence, proactive interruption of workplace violence occurrence by running the simulation-based behavioral training and behavioral emergency response teams are required. Also, individual-level approaches of clinical screening program combined with a psychiatric counseling clinic, that allows for training physician visits during the daytime and provides pharmacotherapy and cognitive behavioral therapy, are needed.

## Data Availability

Participant-level data is not publicly available, but can be accessed by researchers who meet the criteria for access to de-identified sensitive data via request to the corresponding author (issac73@snu.ac.kr), under the term of clinical research ethics committee of Seoul National University College of Medicine and Hospital.
